# Supplementary figures and images for: Forecasting severe grape downy mildew attacks using machine learning
Source: PLoS One. 2020 Mar 12;15(3):e0230254. doi: 10.1371/journal.pone.0230254 (PMC7067461; doi:10.1371/journal.pone.0230254)

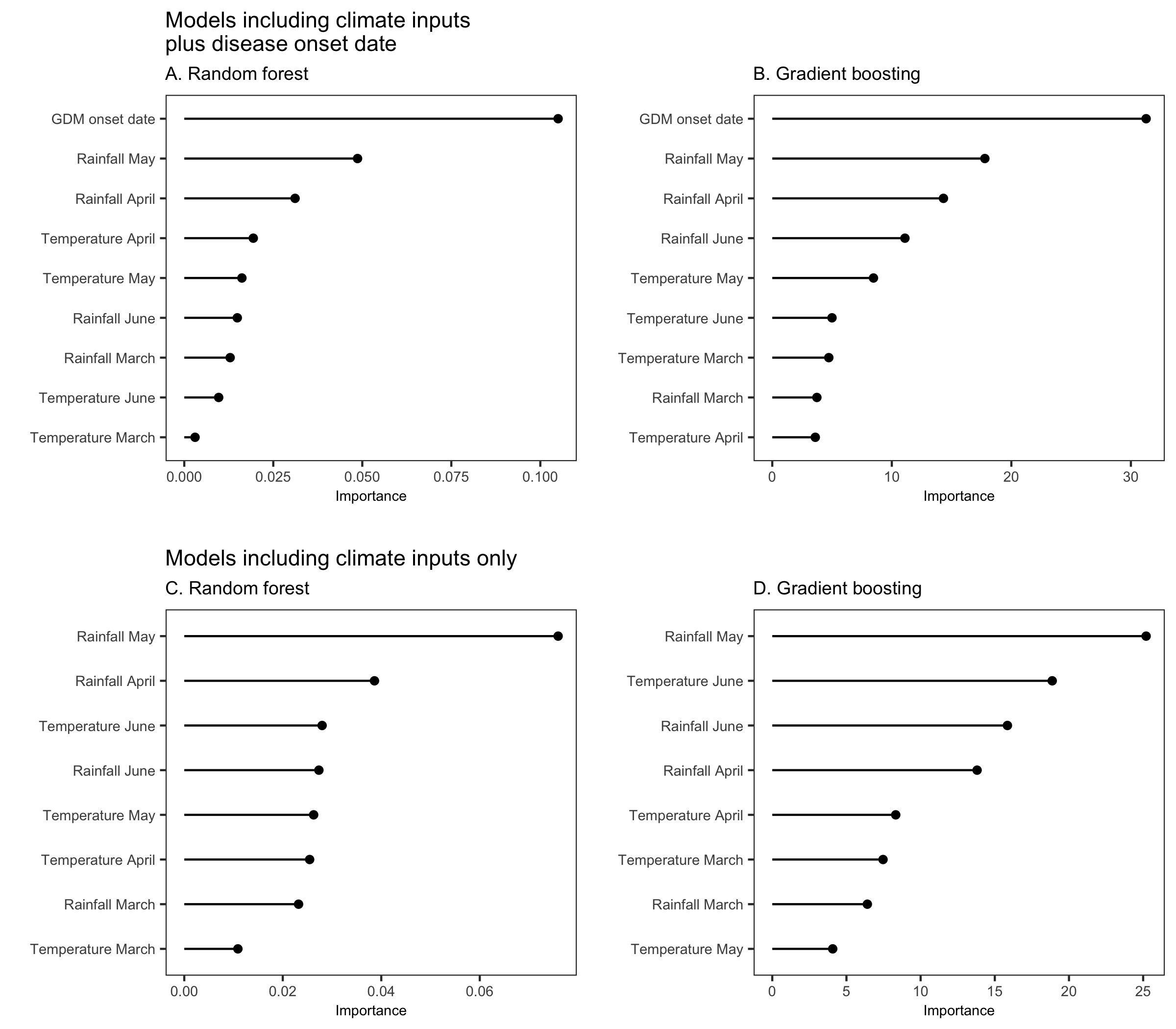

Supplement: S1 Fig — Grape downy mildew (GDM) incidence data on leaves after bunch closing (A) and imputed disease onset dates (B) in 151 untreated plots. Median contamination levels and median disease onset date are represented by vertical dotted lines. (PNG) [file pone.0230254.s002.png]

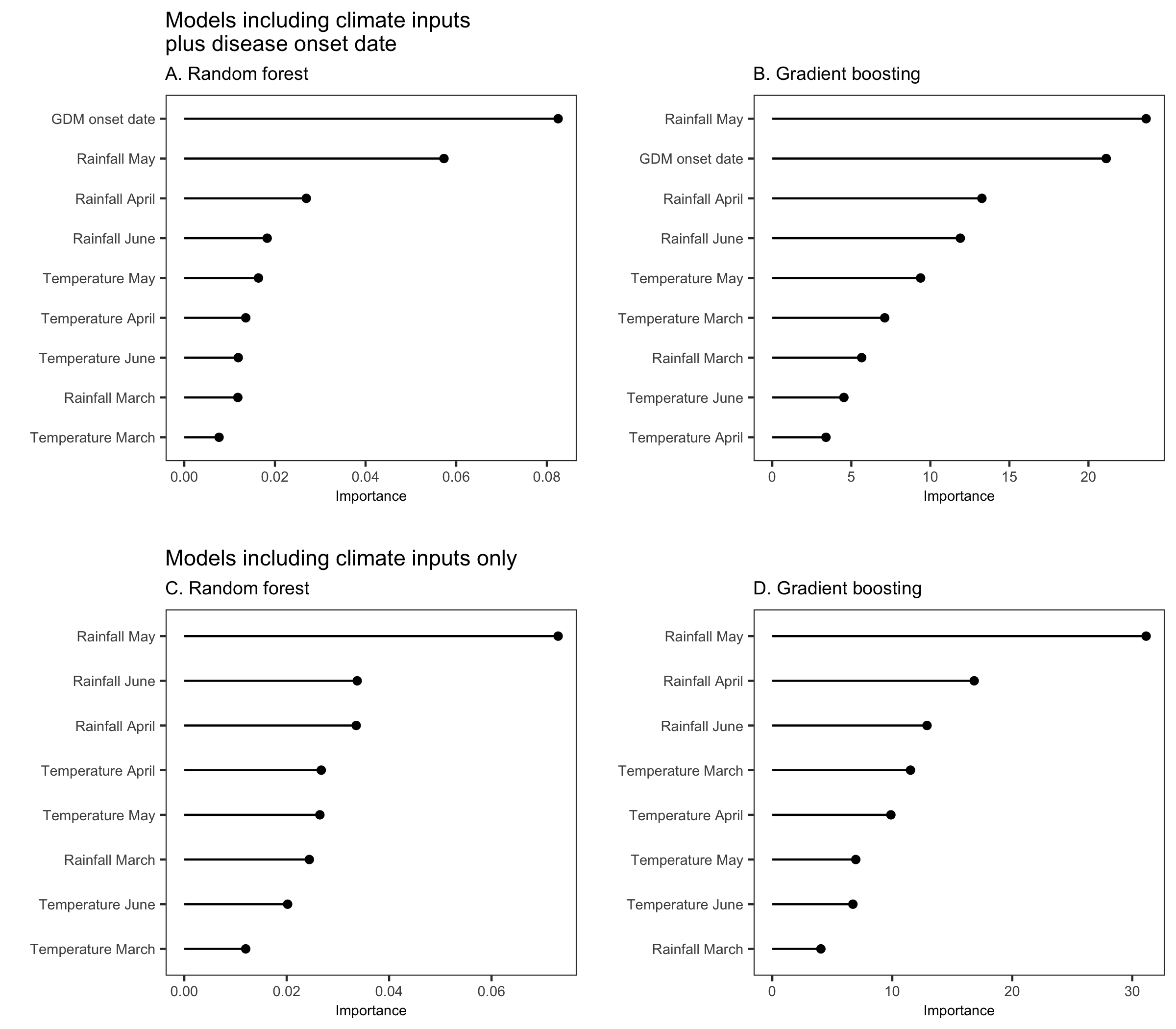

Supplement: S2 Fig — Grape downy mildew (GDM) incidence data on bunches after bunch closing (A) and imputed disease onset dates (B) in 156 untreated plots. Median contamination levels and median disease onset date are represented by vertical dotted lines. (PNG) [file pone.0230254.s003.png]

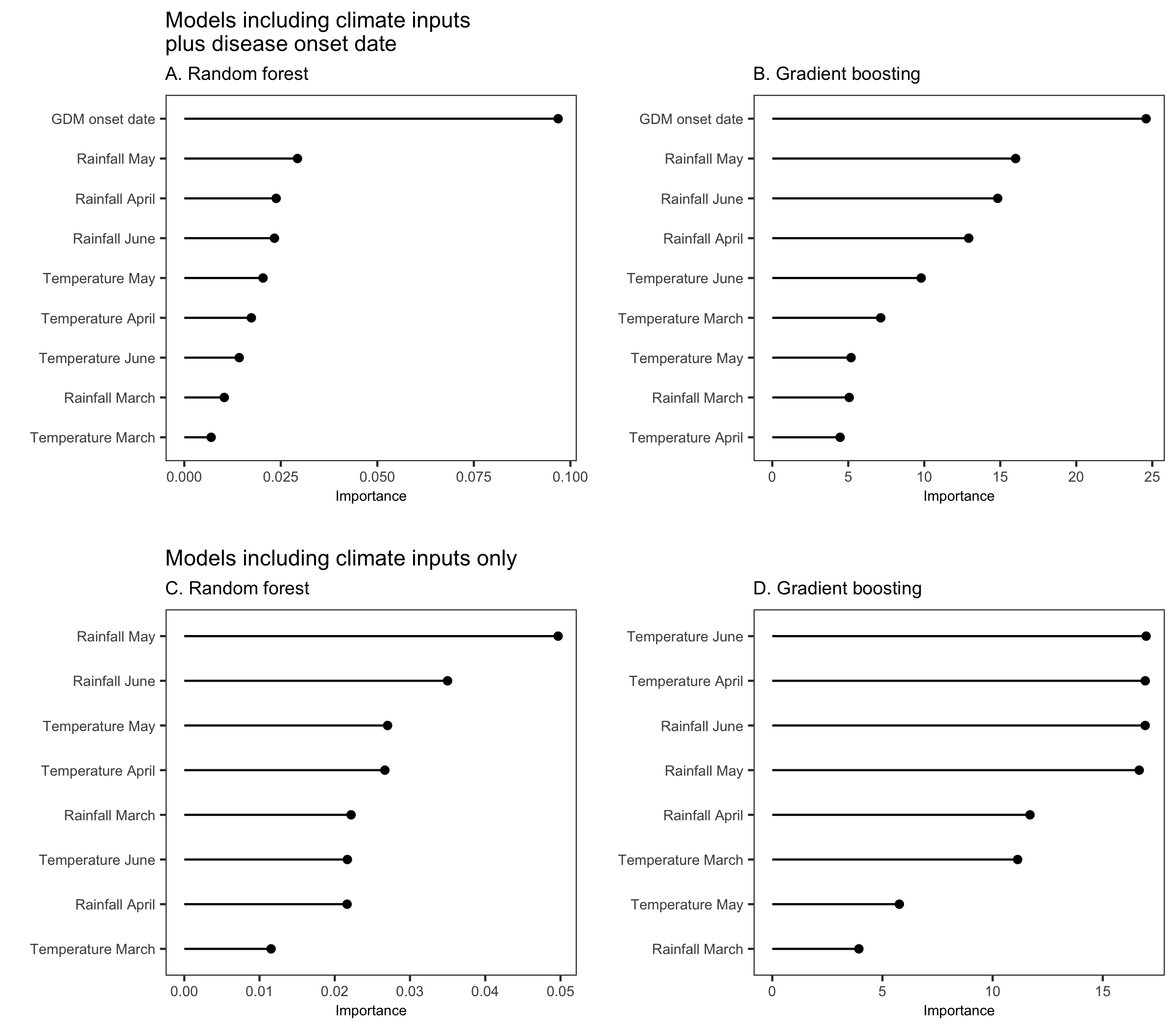

Supplement: S3 Fig — Grape downy mildew (GDM) severity data on bunches after bunch closing (A) and imputed disease onset dates (B) in 152 untreated plots. Median contamination levels and median disease onset date are represented by vertical dotted lines. (PNG) [file pone.0230254.s004.png]

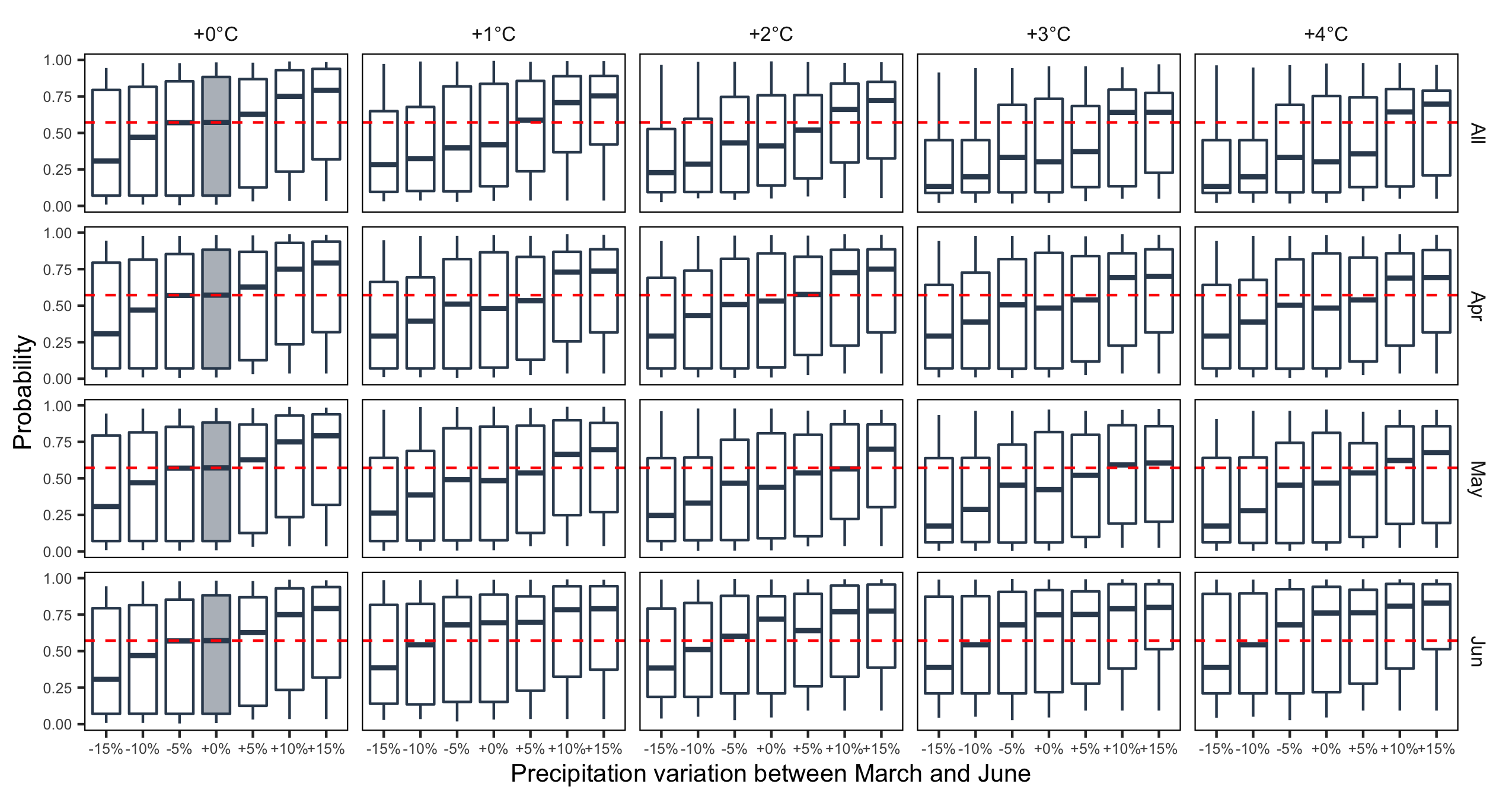

Supplement: S4 Fig — Each graphic shows the effect of precipitation change during a given period (from -15% to +15% between March and June, in April, in May, or in June) for a fixed level of temperature increase (from +0°C to +4°C between March and June) on predicted probability that GDM severity on leaves will be higher than regional median at the end of the season. Probabilities are forecasted by a gradient boosting algorithm that includes all climatic features. Each boxplot represents the distribution of the probability values over the vineyard plots of our dataset; the shaded boxplot corresponds to initial precipitation and temperatures (precipitation and temperature kept unchanged compared to actual conditions) and the median probability obtained with this scenario is indicated by a red dotted line. The lower and upper hinges of the boxes correspond to the first and third quartiles (the 25th and 75th percentiles) and vertical segment represent the range between min and max values. (PNG) [file pone.0230254.s005.png]

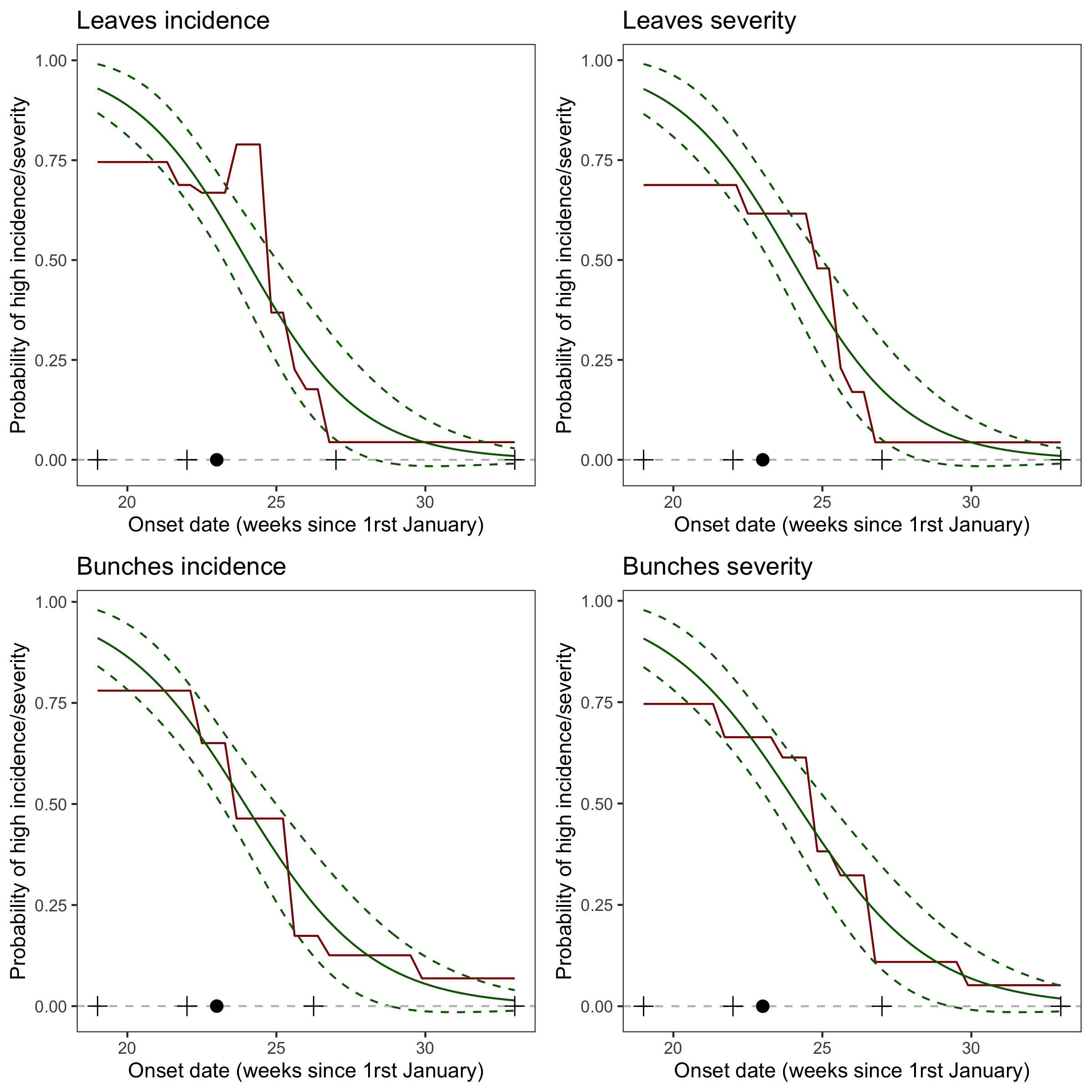

Supplement: S5 Fig — Median, minimum, 1st and 3rd quartiles, and maximum of observed onset dates are represented by a dot and four crosses, respectively. (PNG) [file pone.0230254.s006.png]

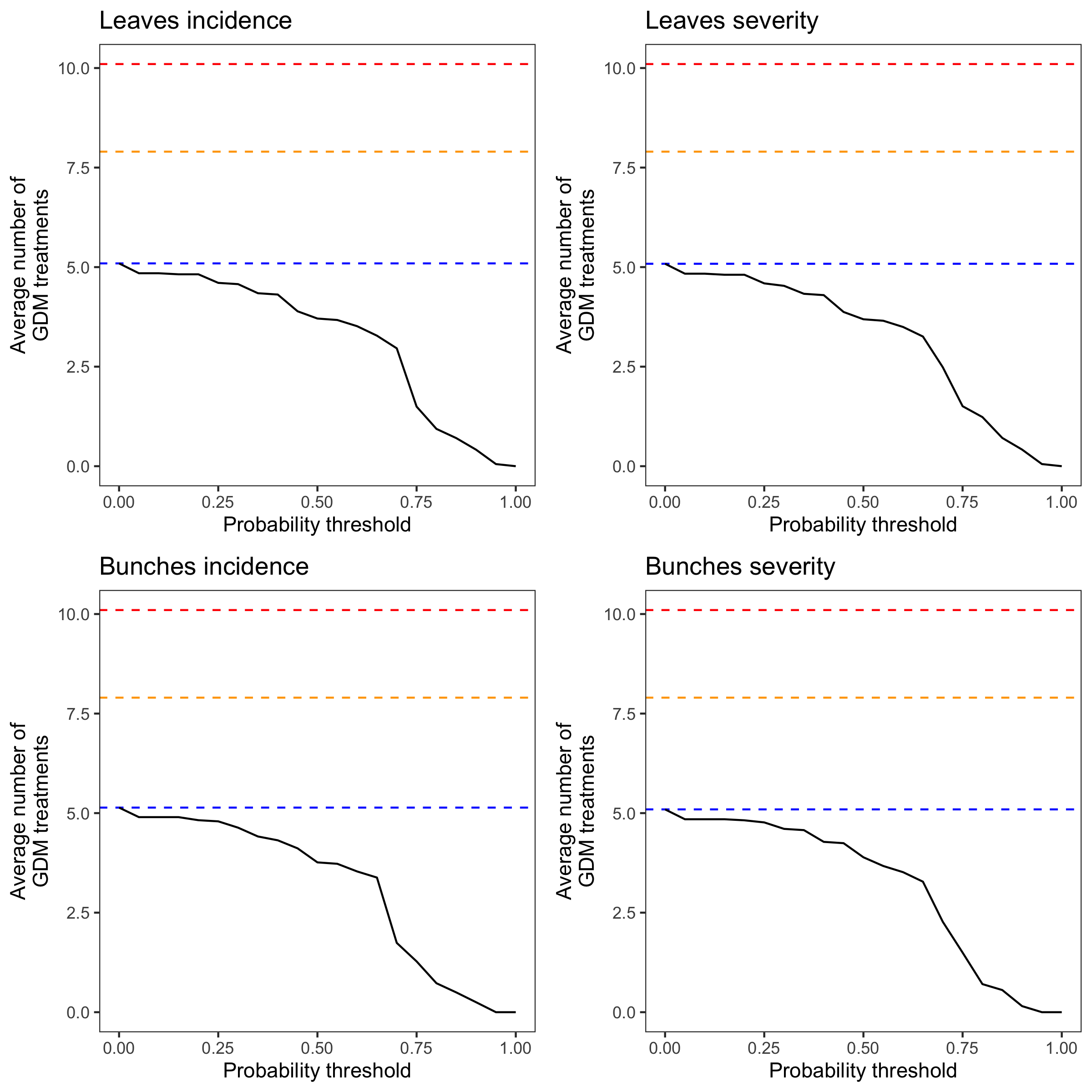

Supplement: S6 Fig — The black curve indicates the average numbers of fungicide treatments in the vineyard plots of our dataset computed while assuming that the first treatment is triggered only when the GLM probability of high severity exceeds the value given in the x-axis. Blue line represents the number of treatments for threshold = 0, i.e. when the first treatment is applied in all plots as soon as GDM symptoms are detected. Red and orange lines correspond to the average numbers of treatments recorded by the SSP in 2013 and 2010, respectively. (PNG) [file pone.0230254.s007.png]
